# The Viral Spread of Health Care Financialization: Big Finance, Big Data, and Big Law

**DOI:** 10.1017/amj.2025.10071

**Published:** 2025-07

**Authors:** Barry R. Furrow

**Affiliations:** Drexel University Thomas R. Kline School of Law, Philadelphia, PA, USA

## Abstract

This reflection article examines the trajectory of health law — using scholarly work by George Annas, Wendy Mariner, and Fran Miller as a platform.^1^ These three health law scholars have been analyzing the complications of health law in the U.S. economy for decades, and each of them has been prescient in anticipating what the future of health care delivery will look like and how we might improve it.

## Introduction. Mining for Gold in Health Care Scholarship

This reflection article examines the trajectory of health law — using scholarly work by George Annas, Wendy Mariner, and Fran Miller as a platform.^1^ These three health law scholars have been analyzing the complications of health law in the U.S. economy for decades, and each of them has been prescient in anticipating what the future of health care delivery will look like and how we might improve it.

George Annas has written hundreds of articles and dozens of books, a corpus too large to summarize here. I will focus on his book *Some Choice: Law, Medicine, and the Market*, in which Annas spends two chapters looking at the problems created by one of the early attempts at reforming health care costs — the use of managed care organizations to walk the medical tightrope between quality patient care and cost-conserving modes of medical practice. This market model went into hyperdrive as health care financing via Private Equity firms began to take over hospitals, nursing homes, and other health care entities. Annas also offers a possible metaphor as a model for health care reform in the future — his idea of an Ecological metaphor.^2^ I will consider this metaphor strategy to refocus on a patient-centered system.

Fran Miller has done extensive and valuable comparative work, looking, for example, at the goal of accountability in health care in both Great Britain and the United States. She argues that new technologies — now taking the form of AI chatbots and other powerful diagnostic tools — “have been a primary catalyst for the burgeoning ‘accountability movement’ in medicine.”^3^ Miller observes that “computer-facilitated aggregation of clinical data tends to raise quality and cost questions which cannot reasonably — or morally — be ignored by any thinking person.”^4^ I foresee benefits and troubles ahead with uses of AI, and I will dwell on the troubles throughout this article.

Wendy Mariner — in her writing on the Affordable Care Act, its passage, and constraints imposed by the Supreme Court — looked at the gnarly legislative features of the Affordable Care Act and the limited understanding of health insurance exhibited by both lawyers and judges. She posits that legal education has failed to train lawyers in a field as complicated as health law, as lawyers have failed to understand core features of health care and missed a chance to develop a better narrative for reform. I will expand on Mariner’s challenges to legal education, asking what we need to do to better train lawyers as dealmakers, regulators, and citizens in order to challenge many aspects of the flawed financing models in our health care system.

My goal is to highlight these three academic stars of the health law firmament and build on their themes in developing a critique of today’s health care system, which has come to involve Big Finance, Big Data, and Big Law. Annas saw the market coming on strong — becoming a dominant metaphor for Big Finance and financialization in health care. Miller anticipated the role of Big Data tools like machine learning and artificial intelligence. Mariner understood the failures of legal education in educating students about the nature of health care and its context, anticipating Big Law and its effects on lawyers. I want to acknowledge their foundational work and take it one step further.

## I. George Annas: Spinning Metaphors to Rethink Health Law

Health Law is a complex and ever-evolving tapestry of medical treatments, diverse health care providers, and financing mechanisms, such as private equity, that have driven rapid privatization of most elements of the U.S. health care system. We find fumbling attempts by the federal government to regulate the side effects of such financing harms, often to very little effect. The Federal Trade Commission (“FTC”) regulates competition and behaviors that raise costs to consumers. The Centers for Medicare and Medicaid Services (“CMS”) is a reluctant regulator that has used economic tools to induce preferred behavior by hospitals in many ways but which shares with the states the power to inspect and regulate nursing homes. They are no match for well-funded companies that move fast, roll up firms to create networks, and sell off facilities before regulators can inspect and act.

We need to rethink the very nature of health care delivery and what drives our current tolerance for market-driven models of care. Metaphors are a useful tool for creating a narrative for thinking about a complex issue.

### A. Why Metaphors Matter

[Metaphors] are inescapable, not merely in language but in thought and action. They are used to comprehend experiences, events, and activities, to understand and “make sense of phenomena in the world in human terms.” Second, metaphorical structuring of concepts and experiences has normative implications. Imagining public health as the fight against microorganisms introduces a discourse of heroes, villains and victims, as well as directive policies of containment, controllability and surveillance, prioritizing the public interest over individual decision-making. Interpreting healthcare as a market and care provision as a business promotes efficiency, competition and profits as primary values, The third characteristic is that metaphors are continuously changing; and perhaps they must. — Henk ten Have & Bert Gordijn, *Metaphors in Medicine.* ^5^

Health care uses various metaphors or narratives to try to capture legal and ethical narratives, and George Annas was a master of metaphor. He was an ethical watchdog with sharp teeth, a bioethicist and social critic, and an astute observer of the forces and cross currents buffeting healthcare over the past fifty years. Medicine began as an ethical universe bound by self-regulation and the ethics of the Hippocratic Oath (along with civil law medical malpractice suits, much loathed by physicians). Physicians gained power as new drugs, devices, and treatments developed, health care cost inflation increased, and institutions like insurance companies and hospitals began to focus attention on how expensive medical care could be. The passage of Medicare in 1965 began to drive higher prices in the health care system, as did the light regulation of physician fees by the private insurance market. By 1970, federal costs had shot upward.^6^ The response was the HMO Act of 1973, aimed at creating new entities known as Health Maintenance Organizations (“HMOs”).^7^ Medical controls shifted from professional self-regulation to directly incentivizing doctors to save money for the insurance company (while not hurting quality). HMOs put physician income directly at risk for the first time.

**Health Care as War**. Annas has written about all of this.^8^ He has offered two metaphors capturing stages of health care evolution. The first is Health Care as War.^9^ Annas described war against disease as a common and powerful metaphor to justify medical diagnoses and costly procedures.^10^ The doctor’s job is to diagnose diseases and kill them. For doctors dealing with deadly diseases, or public health officials, the war metaphor is appealing, making physicians gladiators fighting aging to the bitter end, curing cancer, obesity, and other acute and chronic diseases. But it has limits and may be counter to good medical practice. The negatives are well-described by Paul Hodgkin: “it emphasizes that taking action is a virtue, patients are passive, the main protagonists in this drama are doctors and diseases (patients are not the ‘real’ focus), technologies are weapons (and thus, implicitly, the more the better), and we doctors know best as we are the ones in control … . For many specialties, however, including geriatrics, psychiatry, and general practice, using the ‘medicine is war’ metaphor can be counterproductive.”^11^ He concludes that the war model is too masculine, stressing control and pursuit of power.^12^ He asks that other metaphors be tested, such as “medicine as collaboration.”^13^

**Health Care as Market**. The market metaphor became the next narrative that came to dominate institutional care — namely, care provided in hospitals, nursing homes, hospices. Annas observed that patients become consumers or purchasers, and health care professionals — and the calling to cure and care for the sick — became yet another market opportunity for profitable service and product promotion and exploitation. Annas writes:

The market metaphor leads us to think about medicine in already familiar ways: emphasis placed on efficiency, profit maximization, customer satisfaction, ability to pay, planning, entrepreneurship, and competitive models. The ideology of medicine is replaced by the ideology of the marketplace. Trust is replaced by *caveat emptor.* There is no place for the poor and uninsured in the market model. Business ethics supplants medical ethics as the practice of medicine becomes corporatized. Hospitals become cost centers, as nonprofit medical organizations tend to be corrupted by adopting the values of their for-profit competitors. A management degree becomes as important as a medical degree. Public institutions, by definition, cannot compete in the for-profit arena, and risk demise, second-class status, or privatization.^14^

An apt description. As explained in the words of one reviewer, Annas lays out the limits of the market thusly: “The market contains neither the commitment nor the constraint of the Hippocratic ethic. Positive market attributes such as efficiency and customer satisfaction can benefit retail businesses, but patient care and rational institutional deployment of scarce resources is harder in health care.”^15^ Amen to this observation. This leads Annas to search hopefully for another metaphor for a new better stage in our health care system. He proposes the ecology metaphor, which Annas associates with such words as “integrity, balance, natural, limited (resources), quality (of life), diversity, renewable, sustainable, and stewardship.”^16^

I will return to metaphors later. First, I want to update Annas’ critique of the market.

### B. The Market as Metaphor

Financial institutions, such as venture capital and private equity firms, typically use “three magic words to justify their investments” in health care institutions (*e.g.*, nursing homes, hospitals, and hospice care).^17^ These justifications are the “marketing gospel” for such firms.^18^ They are *markets, innovation*, and *efficiency.* ^19^ The economic claims build on three virtues that are *assumed* to be true, as economists love to do. These elements of economics are often fueled by ideological thinking — devout beliefs undisturbed by empirical evidence to the contrary.^20^

1. *Markets.* The idea of a market economy is grounded on a series of assumptions. Individuals can own and profit from private ownership of business and property. They are motivated to sell to the highest bidder while still paying the least for their goods and services. Competition is universal, keeping prices fair and production efficient. Information about goods and services is perfect. Government’s limited role is to police the market and block the creation of monopolies.

Free market capitalism is an ideology that contains beliefs and values, much like religious fundamentalism, insulated from critique or closer examination of the dominance of the metaphor of the market in our culture. Market ideology has justified the expansion of private finance into health care.^21^ This ideology developed early, for example, in nursing home history in the United States, allowing nursing homes to become an industry built on “market” solutions to end-of-life care for the elderly.^22^

2. *Innovation* as a virtue was the second example of magic thinking in health care ownership. *Innovation* is the first virtuous claim of the financial industry in health care takeovers. I have found “[o]ne website [which] places innovation in a central place in private equity investing: ‘Private equity funds are expected to increase the efficiency and revenues and increase the profits of portfolio companies due to the assimilation of innovation and technology.’”^23^ Another posits “[h]ealthcare is at the forefront of innovation and technology. There is tremendous interest in investing in the healthcare ecosystem to help drive better outcomes — to invest in companies that are innovative and are leveraging technology to create better patient outcomes at a lower cost.”^24^

Innovation as a rationale for investment is powerful in technology-heavy industries, for example, but it lacks justification in nursing homes and hospices. In much of health care, patients are vulnerable, requiring staffs able to give sufficient individual attention. In nursing homes in particular, “innovation and new technologies are not easy to imagine and create in [a] world of the vulnerable and fragile patient” who needs intensive direct supportive nursing care from trained staffs.^25^ Neither innovation nor technology is likely to help in obtaining good staff to give patients the care and respect they deserve.^26^

*3. Efficiency* is the third magic word, promising “that a [health care] facility can work better if it is streamlined” — staff is reduced, procedures are simplified, new vendors are procured at lower cost.^27^ The problem is that hospitals and nursing homes “are not mass production facilities that benefit from speeding up the production line; nor are they complex operations that benefit from modernizing computer systems to eliminate slowdowns in a system — they are utterly human-centric care systems.”^28^ They need well-trained, caring staff to care for patients and produce excellent care for them.

Ultimately, “[p]rivate equity owners achieve neither higher quality nor lower cost” in health care.^29^ I have argued that “[m]arkets are characterized by transparency if prices are public and all relevant activity is visible to everyone.”^30^ Additionally, buyers must have perfect information and be able to shop among interchangeable products.^31^ This fictional market also “assumes traders who engage with one another at a designated time and place, abiding by shared rules — a town square on market day.”^32^

However, “[w]hat we see with private equity health care entities is an antimarket — opaque, monopolistic, with consumers who are unable to shop easily and are often constrained by location.”^33^ Private equity firms prioritize the “concentrat[ion] [of] power and wealth, promising exceptional profits only to a few.”^34^ This is what the French historian Braudel has referred to as a type of jungle capitalism, in which “the great predators roam and the law of the jungle operates.”^35^

### C. Privatization: Private Control

Privatization became a universal solution, as evidenced by the staggeringly long list of services targeted for transfer to private control by the President Ronald Reagan’s Commission on Privatization—public housing, federal loan programs, air traffic control, education vouchers, the Postal Service, prisons, Amtrak, and Medicare, just to name a few. The vision was enormous and comprehensive. It really was the privatization of everything. — Donald Cohen, *The Roots and Reasons of Privatization.* ^36^

Gardner and Scheffler describe privatization as “a philosophy of government that advocates a greater role for private market incentives and the mechanisms of competition in achieving public purposes.”^37^ Those in favor of “outsourcing services to the private sector argue that financial accountability compels private companies to ensure patients’ wellbeing, seek innovation, and eliminate unnecessary bureaucracy.”^38^ According to some, “[t]hese profit motives are then supposed to give private firms a competitive advantage over the public sector, which is often constrained by rigid cultures, regulations, and few incentives to innovate.”^39^ Donald Cohen points out that in the United States, privatization was the path conservatives seized to create “the smallest government possible.”^40^

Early proponents of privatization “argued that a private ownership system could be more efficient — providing quality services at a lower cost.”^41^ Privatization often involves contracting-out specified functions, with the publicly funded service provider retaining decision making power while delegating the provision of certain services to private organizations.^42^ Privatization promised reduction of unnecessary bureaucracy while promoting innovation. This was to be an improvement over the public sector, which is often seen as stagnant and inefficient.^43^

A recent Lancet study of privatization in health care concluded that “[a]t the very least, health-care privatisation has almost never had a positive effect on the quality of care. But outsourcing is not benign either, as it can reduce costs, but seems to do so at the expense of quality of care. Overall, our Review provides evidence challenging the justifications for health-care privatisation and concludes that the scientific support for further privatisation of health-care services is weak.”^44^

The United States has hardly been immune to the forces of privatization. Leslie King writes: “In instituting Medicare and Medicaid, the U.S. did *not* create a system of government-run nursing homes or specifically incentivize the development of not-for-profit nursing homes, as it might have done. Purposefully or not, lawmakers created a system of large, for-profit, chain nursing homes.”^45^

Privatization has been the first step. Financialization has been the second step, as large venture capital and private equity firms have moved through modern economies, rolling up businesses, extracting profits for investors, and too often weakening the entities acquired.

### D. Financialization: PE Plunderers **^46^**

In the popular imagination, private equity is often portrayed as a vulture, or some other scavenger that feasts on the sick and dying. Gross but unavoidable. But the bulk of the work done by modern-day private equity firms is not to finish off-sick companies, but rather to stalk and gut the healthy ones. This type of predation is the result of 50 years of policies that have prioritized the profit-making of a few over the wellbeing of many: a corporate world that grew accustomed to valuing shareholders over everyone else, a penchant for siding with executives over unions, and a legislative establishment loath to enact strict regulations on the financiers whose donations fuel their campaigns. In short, a toxic soup of regulatory inertia and corporate greed. — Hannah Levintova, *The Smash-and-Grab Economy.* ^47^

Financialization is the next stage in both our retail and health care economy.^48^ It refers to the “increase in size and importance of a country’s financial sector relative to its overall economy.”^49^ Privatization of health care services is already underway across nursing home systems, hospitals — particularly in rural areas — physician groups, dentistry, and many other health care providers. Examples of financialization include the move by big financial firms into recently privatized businesses or the selection of other tempting areas in health care, such as behavioral health and eating disorders, with room for acquisitions on behalf of their investors. The most recent development over the past two decades has been the move of private money away from venture capital and other sources of private dollars to private equity firms, with their supercharged incentives for large and quick profits at little risk to the investors and much higher risk to acquired institutions.^50^

Private equity firms have moved into the ownership of hospitals, nursing homes, hospitals, and physician groups.^51^ These firms pool money from sophisticated investors, ranging from wealthy people to college endowments and pension funds, and use that money to buy and flip at a large profit within three to seven years. According to one source, private equity (“PE”) “has poured nearly $1 trillion into nearly 8,000 health care transactions during the past decade.”^52^ PE claims to address inefficiencies and revitalize failing firms. Critics see a far more negative picture, noting that “private equity’s playbook, while it may work in some industries, is ill suited for health care, when people’s mental health and lives are on the line.”^53^

This financialization of care is shaped by profit maximization, often aimed at finding tools to maximize government reimbursement from Medicare and Medicaid through the use of price inflation and quality reducing tools such as staff reductions. Private equity financing of significant parts of this spectrum of care redefines the patient from a vulnerable person to be cared for to a passive magnet for reimbursement dollars. Appelbaum and Batt note that:

[H]ealthcare financialization has occurred along two parallel tracks: from ‘the inside out’ – as nonprofit hospitals increasingly adopt non-healthcare-related financial strategies to survive; and from ‘the outside in’ – as financial actors have moved into healthcare because they view it as a lucrative investment. Nonprofit, for-profit, and private equity owned hospitals have contributed in different ways to the process of financialization in healthcare – in which the logic of financial calculations often overshadows the logic of human care giving.^54^

Consider the tools of PE financing of health care institutions and their effect on health care institutions. PE offers a short time horizon, typically three to five years, and often promises a rate of return of around twenty to thirty percent.^55^ After three to five years, the PE firm moves on, having extracted maximum value from the target firm and leaving it in poor condition.^56^

PE firms utilize leveraged buyouts (“LBOs”) and Real Estate Investment Trusts (“REITs”) as core parts of their strategy. An LBO is “the acquisition of one company by another using a significant amount of borrowed money” to meet acquisition costs.^57^ The target firms assets are “often used as collateral for the loans along with the assets of the acquiring company.”^58^ In such a transaction, “the ratio of debt to equity used for the takeover will be as high as possible,” using the target company’s assets as leverage.^59^ This is a tool for plundering the acquired firm by through the use of debt — it allows PE firms “to pay themselves and other shareholders dividends in the order of magnitude of hundreds of millions of dollars.”^60^ This asset-stripping is the mark of private equity ownership of hospitals and nursing homes in particular.^61^ This debt burdens the acquired company and little, if any, of this money contributes to care improvements.^62^ The target entity is often destabilized by this process.^63^ Many target companies experience bankruptcy as a result.^64^

Staff reduction programs are another “market” tool to cut costs, used aggressively to enable firms to hit their rate-of-return metrics.^65^ As I have written elsewhere, “[c]utting [staff,] billing, legal, and human resource departments reduces ‘wasteful’ overhead…[,] draining nursing home assets” while leaving institutions short of caregivers.^66^ Reducing staffing is also followed by a change in the patient mix. Recent studies of privatized hospitals found that such hospitals “tended to search for more financial efficiencies by targeting more profitable patients and by reducing the levels of staffing.”^67^ As hospital owners change their patient mix, “the intake of low-income Medicaid patients disproportionately declines,” reducing aggregate Medicaid volume.^68^

Related-party transactions are a third tool to skim the till of health care facilities. The goal is to create “new financial streams [through] complex ownership and management structures.”^69^ As part of this strategy, “[n]ursing home chain owners often outsource goods and services to multiple entities in which they also have ownership, and these entities then allow ancillary clinical services to be fragmented and spun off under the corporate umbrella.”^70^ They then “skim the till” — the “owners can arrange highly favorable contracts in which their nursing homes pay more than they might in a competitive market.”^71^ As a result of these contracts, “[t]hese profits are then siphoned off, not recorded in nursing home accounts, and hidden in affiliated companies.”^72^ This is fraud on the federal government.^73^

The complex layers of corporate entities in one health care system “also shield the enterprise from liability by making it difficult for plaintiff lawyers to pursue a defendant with assets.”^74^ These “strategies often involve deception in financial statements to conceal real income and assets among a myriad of subsidiaries.”^75^ Lacking “data and visibility, plaintiff lawyers and regulators are all blinded.”^76^

We will return in Part V to consider regulatory approaches to controlling private equity ownership in health care or, perhaps, banning it altogether.

## II. Fran Miller: Rethinking Provider Accountability

### A. The Coming of Big Data and AI tools

Miller’s work has looked at how modern information technology can link to accountability in the health sector.^77^ Health service databases about providers that anyone can access creates “a powerful stimulus to health care quality improvement.”^78^ Her study of Britain, the United States, and Australia, leads her to the conclusion that this technology not only aids “third parties in monitoring, improving and policing the quality of care”,^79^ but “US experience demonstrates that information technology turns out to be an equally powerful stimulus to provider self-improvement and quality innovation.”^80^ Miller concludes that “making the fruits of information technology more widely accessible to all can substantially improve overall patient health.”^81^

Aggregation of clinical data exposes ineffective therapies, raising cost and quality issues. It forces a hard look at whether better modes of treatment are available. She writes: “[a]ccountability and health care information technology thus fit hand in glove to further the interests of better overall patient health services. At its best, the law functions as handmaiden to their symbiotic relationship.”^82^ Public disclosure of medical data helps consumers and buyers to see where value lies and to compare institutions and doctors. Consumer/purchaser choice of health care is a tool of a market-driven health sector. The goal is greater accountability for doctors and institutions in the delivery system. Physician profiling, revelations of medical practice variation across the country, and hospital comparisons have led to government databases turned into website comparison sites. The academic writing in the 1990s began to look at the value of outcome statistics in comparing providers.^83^

### B. Tracking Outcomes in Institutions

Miller’s work proved prescient.^84^ Accountability in today’s health care environment depends on regulatory strategies that compel accountability in a privatized health care system. “Performance monitoring is required,” for example, “by the Joint Commission, which issued standards in 2010 on medical staff governance to prescribe the relationship between the medical staff, the medical staff’s Executive Committee, and the hospital’s Board.”^85^ Joint Committee “standards intensify prospective monitoring of physician quality; one of the standards, for example, specifically provides that the hospital must establish a system for collecting, recording, and addressing individual reports of concerns about individual physicians.”^86^

The Joint Commission is a private not-for-profit organization that accredits hospitals, nursing homes, and other health care entities.^87^ It has been aggressive in developing standards for hospitals that requires levels of evaluation of providers, including “a period of focused review for all new and all renewal privileges for existing providers.”^88^ This “standard requires the medical staff to develop criteria for evaluating the performance of practitioners.”^89^ The standard “also includes examples of triggering events, such as ‘infection rates, sentinel events, complaints, or other events that are not sentinel events.”^90^ Data is required to achieve effective compliance of the medical staff in hospitals.

The value of data analytic tools for gathering this mandated information through machine learning is clear, since spotting triggering events is what data tools are best at. “Hospitals are increasingly under pressure from multiple sources to track and prevent adverse event creation,”^91^ as I have discussed above.^92^ Federal requirements have also changed hospital-staff relations. For example, under the Medicare Conditions of Participation, federal law requires among other things that hospital bylaws reflect the accountability of the medical staff to hospital governing board or “governing body for the quality of care provided to patients.”^93^

### C. Forcing Accountability through Transparency

Accountability is vastly enhanced by the tools of machine learning and AI tools. We demand it in hospitals by linking CMS reimbursement of Medicare dollars to the lowering of patient readmission rates, infection rates, and adverse events of specific kinds.^94^ “Fix the problem or take a Medicare reimbursement hit,” says CMS to thousands of U.S. hospitals.^95^ This reimbursement-driven accountability forced on hospitals has drawbacks as well as benefits, forcing hospitals to pay attention to CMS metrics.^96^ CMS has become a regulator through the power of its purse — as Eleanor Kinney wrote, “Medicare eventually had to become a procurement program and finally a regulatory program. Because of the inflationary costs and charges presented by providers and suppliers for compensation, the Medicare program had to resort to rate regulation to control Medicare expenditures.”^97^

The next step is the use of AI to improve medical practice by physicians and hospitals. The problem is that take-up of AI has been slow by health care institutions such as hospitals. The costs of hardware, software, updates, maintenance and the need to hire trained staff may explain this slow take up of AI systems.^98^ Doctors may worry about liability if a patient is harmed as a byproduct of AI use. Legal rules may not be fully developed or sufficiently clear. They may be afraid of deep learning, difficulties in explaining results to patients, fear of job loss, or fear of dehumanization of the care relationship. Public agencies may also be slow to regulate out of fear of the new technologies and their costs.

What about AI use by financial firms continuing their harvesting of the assets of health care firms? The literature on AI adoption is now robust as to both pros and cons. Nonetheless, AI take up by private equity is in full bloom.^99^ AI can run thousands of scenarios for how to maximize extraction of value from a health care system. Can regulators keep pace? Can lawyers who represent patients match defendants with deep pockets and powerful tools in their hands? Is health care really moving toward a data-driven and patient-centered future? And will it be a good thing?

## III. Wendy Mariner: Challenging Legal Education to Do Better

Wendy Mariner has written about the fight over the constitutionality of several provisions of the Patient Protection and Affordable Care Act (“ACA”).^100^ She concludes that constitutional scholars were often lacking understanding of health insurance and the health care system — this lack of knowledge was a problem in the legal fight over the ACA. Lawyers arguing the range of challenges of the ACA before the courts often had a limited understanding of the underlying health care issues. Mariner argues that this gap suggests a need to improve legal education to train lawyers in the reality of health care and how to frame arguments.^101^ She offers ideas for improving legal education.

Her most telling observations relate to the narrow focus of legal education, which she subjects to the Social Justice Critique. She notes that the usual law curriculum is built around private law with corporate and property issues the primary focus; this is designed to “train students to accept the existing structure of public and private institutions and financial relationships as normative and neutral in their effects, and thus to perpetuate the status quo.”^102^ Her suggestion is to use health law to address distributive justice, noting that “[t]he study of health law can encourage critical thinking about legal approaches in light of their respective civic and ethical implications.”^103^ She then cites Annas, who noted that “studying medical problems provokes thinking about what it means to be human and ‘therefore what rights and obligations humans should have.’”^104^

I agree with this. The damages inflicted by the financialization^105^ of U.S. health care, driven by private equity financing of everything from hospital systems to hospice care, is becoming visible to regulators. Now it needs to be understood by every law school graduate. Why? Because much of this financing is designed to take over existing institutions, strip their assets while overbilling the Medicare and Medicaid programs, and flipping the systems after a few years (or bankrupting them), often leading to bankruptcy of existing once viable systems.^106^ And lawyers doing these deals for financial firms have aided and abetted this stripping of assets from health care entities. They have ethical and fiduciary duties to consider as they take on clients that can do harm in the health care space.

### A. Expanding Both Institutional and Lawyer Fiduciary Duties **^107^**

Nursing home residents are a case study for the expansion of fiduciary duties on to their owners and the lawyers that created the new entities. Residents are uniquely vulnerable. The court in *Schenk v. Living Centers-East^108^* spoke to the particular vulnerability of nursing home residents: “Most residents are vulnerable physically, lack the cognitive skills in many cases to protect themselves, did not ‘choose’ their home, and need a heightened level of personal attention and care.”^109^ Another court “observed that no relationship better ‘fits the description [of the fiduciary capacity] than [the relationship] which exists between a nursing home and its residents.’”^110^

Step one is convincing civil courts to impose a fiduciary duty on an organization such as a private equity firm that invests in hospitals, nursing homes, hospices. Fiduciary law “raises the baseline for conduct and the measurement of failure and breach of duty.”^111^ It can also “create a presumption of wrongdoing for this class of relationships.”^112^ As I have written previously, “Fiduciary law creates legal rights grounded in equity for those who need protection, allowing tolling of statutes of limitation and easing a plaintiff’s burden of proof. And fiduciary law can draw on a range of equitable remedies.”^113^ As noted by Velasco, “[r]emedies in fiduciary law are ‘comprehensive and tenacious.’”^114^ Equity rather than law provides the enforcement engine for breaches of fiduciary duties. Remedies are not limited to equitable compensation, and can “include disgorgement, equitable recission, accounting for profits, constructive trusts, and declaratory judgments—a whole equity arsenal is available.”^115^

Step two is to ask if lawyers are also fiduciaries in private equity deals. Lawyers are heavily involved in doing financing deals in health care with private equity firms that are lucrative clients. If “private equity financing can be ‘immoral’ in the case of private equity financing of health care roll-ups to create new systems of nursing homes, behavioral health facilities, dental clinic networks, and other providers of health services,”^116^ should the lawyers doing the deals also be charged with violation of their fiduciary duties? As I have argued elsewhere, “[t]he lure of high salaries and the pressures of big firm values can corrupt lawyer’s judgment, rationalizing harms to third parties as little more than ‘collateral damage; of an efficient market mechanism.”^117^

We start with the promises of private enforcement. “Fiduciary law is a private law enforcement tool to achieve some form of accountability, as is tort law.”^118^ Hospices, nursing homes, and hospitals may be “appropriate for the use of fiduciary doctrine, which normally tends to focus on corporate dealings with shareholders and other financial situations that involve wrongdoing.”^119^ As I have noted previously, “[f]iduciary duties are common in the corporate world, imposing duties on boards of directors and corporate managers,” and “[f]iduciary duties give the beneficiary of such duties a right to sue the fiduciary for their breach of fiduciary duty, as a matter of private remedies.”^120^ As I have also argued, “[f]iduciary law coupled with a court’s equitable tools provides a far more muscular approach to nursing home bad actors than does either tort or contract law limited to damage remedies. Fiduciary law offers future-looking equitable tools to change the private equity calculus for those who invest in and run nursing homes.”^121^

Fiduciary law aims to identify “opportunistic power used against the vulnerable.”^122^ Private equity owners are the quintessential opportunistic owners under fiduciary law, which “allows vulnerable beneficiaries and their families to demand honesty and selflessness of their fiduciaries.”^123^ Such a fiduciary obligation makes sense for health care institutions, now ruthlessly privatized for gain. Can such a duty also be extended to lawyers who do private equity deals?

#### 1. Primary Fiduciary Duties of Lawyers^124^

The ABA Model Rules of Professional Conduct remind us that lawyers are not only the agents of clients but also licensed fiduciaries with obligations to third parties.^125^ The Rules were first adopted in 1983.^126^ Section (b) of Model Rule 1.6 states what I consider “a lawyers’ minimum fiduciary responsibilities to third parties.”^127^ It states:

(b) A lawyer *may* reveal information relating to the representation of a client to the extent the lawyer reasonably believes necessary:

1. to prevent reasonably certain death or substantial bodily harm;
2. to prevent the client from committing a crime or fraud that is reasonably certain to result in substantial injury to the financial interests or property of another and in furtherance of which the client has used or is using the lawyer’s services;
3. to prevent, mitigate or rectify substantial injury to the financial interests or property of another that is reasonably certain to result or has resulted from the client’s commission of a crime or fraud in furtherance of which the client has used the lawyer’s services.^128^

These rules allow lawyers to release information “to prevent death or serious bodily harm, to stop a client from committing a crime or fraud harming the financials of a third party, or to rectify such harms.”^129^ By giving a lawyer this chance, it creates a safety zone for disclosure, acknowledging that lawyers have a fiduciary duty “to third parties to avoid serious harms and they need to consider these duties.”^130^ Rule 1.6 creates a “pathway to tort law duties of aiding and abetting.”^131^

Rule 1.2: Scope of Representation & Allocation of Authority Between Client & Lawyer, also governs the client-lawyer relationship. Section (d) states that “[a] lawyer shall not counsel a client to engage, or assist a client, in conduct that the lawyer knows is criminal or fraudulent, but a lawyer may discuss the legal consequences of any proposed course of conduct with a client and may counsel or assist a client to make a good faith effort to determine the validity, scope, meaning or application of the law.”^132^

Rule 4.1(b) takes the obligation one step further, imposing a disclosure requirement, prohibiting lawyers from failing to disclose material facts to third parties to avoid assisting client in crime or fraud.^133^

#### 2. Tort Law: Aiding and Abetting^134^

I have argued that “accountability is needed for many private equity deals in the health care area.”^135^ Fiduciary doctrine aims to control “opportunistic behaviors that harm vulnerable parties,” and some courts have even adopted “a cause of action for a lawyer’s aiding and abetting her client’s breach of fiduciary duty,” with attendant liability for the lawyer’s law firm.^136^ Both the Restatement (Second) of Torts section 874^137^ and the Restatement (Third) of Law Governing Lawyers section 51^138^ recognize a cause of action for a lawyer’s aiding and abetting a client’s breach of fiduciary duty.^139^ Section 51, comment h reads: “[a] Lawyer is usually so situated as to have special opportunity to observe whether the fiduciary is complying with those obligations.”^140^

Tort law also “offers a second source of duties built on tort law rules based on the responsibility of persons to third parties,”^141^ as articulated by Section 876 of the Restatement (Second) of Torts, which states:

For harm resulting to a third person from the tortious conduct of another, one is subject to liability if he (a) does a tortious act in concert with the other or pursuant to a common design with him, or (b) knows that the other’s conduct constitutes a breach of duty and gives substantial assistance or encouragement to the other so to conduct himself, or (c) gives substantial assistance to the other in accomplishing a tortious result and his own conduct, separately considered, constitutes a breach of duty to the third person.^142^

These tort duties overlap with fiduciary obligations, exposing lawyers to liability risks where third-party harms may result from their actions on behalf of their clients.

### B. Rethinking Legal Training

Our roles as teachers in a law school classroom are changing. Here Wendy Mariner uses her scholarly scalpel to good effect in asking whether lawyers need a broader education in the world of economics and deal making before they make career decisions. Much harm can be done as a result of the financialization of health care. Law students need to understand how the health care economy works, how businesses are financed and how health care has, in many areas, been taken over by private equity.^143^ The *Enron* disaster and other crises can be blamed in part on legal engineering — every law student should learn about how law can not only do good but can also cause damage to third parties.^144^

My law school’s health law concentration requires that students take the school’s Health Care Finance course. As I have previously argued, it would be useful to add a regular lecture series to help law students understand how the mechanisms of financing health care can be highly damaging to patients, physicians, hospitals, and other actors in the system.^145^ Law students need to understand the nature of Big Law today—the ethical dimensions of tradeoffs that are made when representing health care clients and the “power of money in shaping the prevailing ideologies of privatization in health care.”^146^

### C. Improving Legal Services: What Happened to Auto Clubs and Antioch Law School?

If graduates choose not to work for Big Law, or decide to work toward change within large law, they can be a positive force for change. However, we need some law schools that have a public service committee as their core value.^147^ Many law schools have a range of public interest clinics that serve the poor. Perhaps we need to consider more law schools that copy the Antioch model. The clinical education model that served clients without resources was the Antioch Law School in Washington, D.C., created in 1972 by Edgar and Jean Cahn.^148^ They designed Antioch to be both a law school and also a public interest law firm — with professor-lawyers and student-advocates providing pro bono legal services to the indigent and those unable to obtain representation.^149^ Learning and practice were combined and new law students spent their first six weeks (later reduced to two) living with families in some of the poorest neighborhoods in Washington, to better understand their legal needs and practical barriers in their lives.^150^ By the time of its closure in 1988, Antioch had “handled over 10,000 public-interest cases and trained 1,500 public-interest lawyers.”^151^ It was later renamed as the University of the District Columbia David A. Clarke School of Law.^152^

Other alternative models might be built on existing organizations in some form. For example, another model, thriving early in the nineteenth century until destroyed by the organized bar, was a legal service model of automobile clubs in the early 1900s.^153^ These clubs provided free legal services to members, ranging from defense of driving-related crimes to impact litigation to effect policy changes.^154^ The auto club lawyers represented members in tens of thousands of cases before they were prohibited from practicing law with the auto club platform.^155^ The bar launched a campaign against these legal services; the result was the destruction of “a unique and socially valuable mechanism to deliver affordable legal services at scale. It would fundamentally alter the balance of power held by legislatures and courts when it came to defining and policing law practice. And it would plant other doctrinal seeds that eventually sprouted and grew into the country’s current, profound access-to-justice crisis.”^156^ Perhaps legal ethics now has created too many barriers to ideas such as this. Pity.

## IV. Metaphors: The Patient at the Center of Care

What chills my bones is indignity. It is the loss of influence on what happens to me. It is the image of myself in a hospital gown, homogenized, anonymous, powerless, no longer myself. It is the sound of a young nurse calling me, “Donald,” which is a name I never use—it’s “Don,” or, for him or her, “Dr. Berwick.” It is the voice of the doctor saying, “We think…,” instead of, “I think…,” and thereby placing that small verbal wedge between himself as a person and myself as a person. It is the clerk who tells my wife to leave my room, or me to leave hers, without asking if we want to be apart… . That’s what scares me: to be made helpless before my time, to be made ignorant when I want to know, to be made to sit when I wish to stand, to be alone when I need to hold my wife’s hand, to eat what I do not wish to eat, to be named what I do not wish to be named, to be told when I wish to be asked, to be awoken when I wish to sleep. *Call it patient centered-ness, but, I suggest, this is the core: it is that property of care that welcomes me to assert my humanity and my individuality.* — Donald M. Berwick*, What ‘Patient-Centered’ Should Mean: Confessions of an Extremist.* ^157^

Annas prepared the ground for planting metaphors. His preferred metaphor, the ecology metaphor, he associates with “words such as ‘integrity,’ ‘balance,’ ‘natural,’ ‘limited (resources),’ ‘quality (of life),’ ‘diversity,’ ‘renewable,’ ‘sustainable,’ ‘responsibility (for future generations),’ ‘community,’ and ‘conservation.’”^158^ As Annas explained, “[i]f applied to health care, the concepts embedded in these words and others common to the ecology movement could have a profound influence on the way the debate about reform is conducted and on plans for change that are seen as reasonable.”^159^ This moves us to population health among other perspectives.^160^ Annas wrote: “Applied to medicine, the ecological metaphor can encourage an alternative vision of resource conservation, sustainable technology, acceptance of death as natural and necessary, responsibility for others, and at least some degree of community.”^161^

This metaphor has power, building on the emerging ideas of social determinants of health and wellness generally. However, it does not address head-on the magnetism of the market and the seductive charms of its illusions of efficiency and innovation. We need to couple Annas’ ecology metaphor with others that build a strong image of the vulnerable patient, protected by both public and private law, who deserves to receive a patient-centered care of high quality. Annas also wrote powerfully about how to protect vulnerable patients and I will return to this shortly.

Consider the range of metaphors emerging from both medical practice and the academic literature that place the patient first.

### A. Dignity

As noted by Clancy et al., “[d]ignity is a central theme on the political agenda in all four Nordic countries” — Norway, Finland, Sweden and Denmark.^162^ Norway has a regulation called “The Dignity Guarantee” that aims “to ensure that older adults are treated with dignity when receiving health and care services.”^163^ Finland, Denmark, and Sweden have similar dignity policies.^164^ In one study of dignified care in Nordic countries, the authors write: “A person’s health and well-being are essential to living as full a life as possible. Older adults are no exception.”^165^ The study concludes:

These values embrace the right to a dignified life that includes the possibility of experiencing well-being. To experience well-being means to live under secure conditions and to experience an active and meaningful life with others. To live a dignified life entails that social services must be of good quality and that professionals show respect for the older adults’ privacy and integrity. The self-determination, participation and individualisation of older adults must be respected and supported, and caring staff must be responsive and empathetic in their meetings with older adults.

The concept of dignity can be defined as a core value grounded in respect and associated with human rights. Dignity is also a subjective experience related to autonomy and identity. Heggestad et al. emphasise that dignity is not only a theoretical concept but that it has practical meaning and is of importance to older adults, their relatives and healthcare. When experienced in specific situations, dignity seems to be associated with respect, prevailing personal integrity, and with empathic and compassionate caring.^166^

Dignity deserves to be revitalized in our thinking about the vulnerable.

### B. Stewardship

One might ask, what is stewardship? The World Health Organization defines stewardship as “the careful and responsible management of the well-being of the population.”^167^ Government should act as steward, “tak[ing] responsibility for the population’s health by guiding the health system as a whole.”^168^ Governments must pursue “improvements beyond typical public health and purchasing roles, such as providing health insurance to state employees. This means developing a strategic framework for health policies that reaches all citizens, building support among stakeholders, regulating and monitoring health care systems, and using data to improve.”^169^ The problem with stewardship as a useful metaphor is that lacks a clear focus as a tool for improving health care.^170^ Perhaps focus can be sharpened, however. The Urban Institute reported on state government stewardship efforts.^171^ The report focused on five states:

Though state governments already have clear responsibilities in providing health care to defined populations, such as Medicaid enrollees and state employees, stewardship asks these state governments to go further, considering how to build high-quality, efficient health care to all residents. In the five states we studied, governments acted as stewards by implementing reforms including the establishment of new state agencies tasked with championing health care innovation; the implementation of bundled payments and global budgets; and the establishment of accountable care organizations, coordinated care organizations, and regional care collaborative organizations.^172^

This research on state government gets at the heart of the stewardship model, evaluating strategies of goal development and strategic frameworks to achieve them.

### C. Patient-Centered Health Care

Don Berwick’s “rights” model puts the patient “at the center of the health care system.”^173^ Berwick wants a health care system that respects patient dignity as a central focus, similar to the Scandinavian mandates to promote patient dignity. One of the featured scholars at BU’s Health Law Celebration, George Annas, along with Jay Healey, proposed in a 1974 article a new model, the Patient Rights Advocate.^174^ This Advocate give hospital patients voices and protections for their dignity.^175^ Such a Patient Rights Advocate aims to protect a vulnerable poorly informed patient against health care institutional pressures. It is built on a platform of fiduciary obligations of health care providers and institutions to protect.

This scholarship is a bridge between Berwick’s patient-centered health care model and a robust use of fiduciary duties to put patients first in our profit-driven system. The Annas-Healey model needs to be revitalized and implemented in a range of health care institutions.

### D. Fiduciary Duties

All owners of health care facilities should be held to fiduciary duties to protect their patients/residents, or all profits will be disgorged. If the courts can be persuaded to impose a fiduciary duty on private equity owners to put the interests of their wards first, this will allow us to use private law tools to restrain and retrain the extreme plundering tools of private equity. Several “regulatory ideas (including private law as well as state and federal regulatory approaches)” are worth considering as means for “enhanc[ing] the obligations of private equity to act ethically, while disciplining and restraining its playbook of profiteering.”^176^

## V. New Models: Pushing Back on Financialization’s Cruelties: From Speed Bumps to Prohibition

In Massachusetts specifically, private equity greed drove Steward Health Care (Steward)—which operates eight hospitals in the state—into bankruptcy. The company’s problems began in 2016, when company executives and Steward’s then-private equity owner sold the hospitals’ real estate to a real estate investment trust (REIT), saddling the hospitals with extortionate rent payments that ultimately landed Steward in $9 billion of debt. It is past time we hold private equity firms and corporate executives accountable for driving companies like Steward into bankruptcy—and empower regulators to prevent similar crises from happening in the future. — Elizabeth Warren, The Corporate Crimes Against Health Care Act of 2024.^177^

As I discuss below, Elizabeth Warren’s *Corporate Crimes Against Health Care Act* of 2024, quoted above, would have been a good step toward holding PE firms and corporate executives accountable for their deleterious actions across the health care sector.^178^

### A. Policing Bad Behavior: Speed Bumps, Spike Strips, and Radar Guns to Trace Ownership and Fraud **^179^**

The threshold questions is whether we would be better off “prohibit[ing] private investors generally from investing in health care enterprises,” since their investment resources are huge.^180^ A counter to this position is that we have “privatized whole elements of our health care system, from nursing homes to hospices, with hospitals hanging on to their non-profit status just for tax breaks.”^181^ We have historically “tolerated privatization without much effective regulation and policing of the side-effects of private equity.”^182^

The response to this is: enough is enough. The evidence is accumulating that financialization has had substantial negative effects and needs to be better regulated. The government is slow to respond; it requires studies, data, and powerful arguments that push against inertia, budget constraints, and the power of lobbyists in a financialized system. The adverse effects of our system, as well as excessive costs imposed by bad actors, are unacceptable. The results of private equity ownership of nursing homes for example reveals increased 90-day mortality by 2.4 percentage points, or 15% of baseline mortality among Medicare residents,^183^ adding up to more than twenty thousand deaths over the course of twelve years.^184^ The Government Accounting Office (GAO) has identified “longstanding issues with nursing home quality, such as infection control and resident abuse and gaps in CMS oversight.”^185^ Many more studies are now being published showing fraud against our system, patient adverse events, and criminal neglect of patients. It is becoming a drumbeat of negatives produced by private equity ownership in particular.

Even a Harvard Business School professor, Professor Leitz, who teaches private equity courses at Harvard, is sounding the alarm.^186^ Professors Leitz and Song write:

The recent, burgeoning evidence base using large data and rigorous statistical analyses raises a number of red flags about the corporatization of health care. The issues should prompt a robust policy discussion as to how PE firms should invest in health care. The focus should be on where and how they are adding value, and where they are potentially detracting from patient care and/or increasing patient and societal costs (including costs to public payers like Medicare and Medicaid, whose spending is ultimately largely financed by taxpayers).^187^

Several models for regulating the bad outcomes from financialization in much of health care should be considered.

#### 1. Speed Bumps: Vetting the Investment Process

I have argued previously that “[f]ederal pre-merger review should be required of all private equity-backed merger and acquisition practices.”^188^ No minimum dollar value should be required to trigger review.^189^ Of course, “this will require amendment of the Hart-Scott-Rodino threshold of $101 million.”^190^ This review should “be conducted by the Federal Trade Commission (FTC) and Department of Justice (DOJ), and the US Department of Health and Human Services (HHS) should also be authorized to review such activities for quality and access risks.”^191^ Furthermore, “state health departments should have multi-agency healthcare transaction approval processes for healthcare transactions.”^192^ This process should “include state attorneys general, administrative agencies, and stakeholders such as patient advocates and labor organizations.”^193^ Researchers suggest that, to be effective, “state regulators and enforcers need broad pretransaction notice; sufficient time to review transactions using substantive review criteria; the ability to administratively approve, conditionally approve, or block transactions; and the means to oversee conditionally approved transactions.”^194^

#### 2. Spike Strips: Halting False Claims

In addition to the above, “[t]he federal False Claims Act should be aggressively applied to police the actions of private equity firms.”^195^ As I have noted elsewhere, “[f]alse claims enforcement is a fully developed tool in the federal arsenal, for firms that submit false claims for payment to Medicare and Medicaid. Furthermore, during the investigation phase of a false claims matter in which the health care company being targeted is owned by private equity, discovery should be directed at the private equity firm itself — and not just the health care company.”^196^ Additionally, “[t]ort civil suits should be also encouraged against bad actors, applying theories of corporate negligence and fiduciary doctrine.”^197^ Civil causes of action could “have the potential to reach higher in the chain of ownership beyond an individual facility.”^198^

#### 3. Speed Traps: Tracking Deception through Transparency Rules

Spotting abuses requires mandated transparency rules to uncover fast dealing, deception, and fraud. The FTC has issued new rules that impose additional reporting requirements on financial firms.^199^ This is a start. Accountability, Fran Miller style, follows inevitably from transparency. Disclosure of information will help to deter opportunistic behavior by private equity firms.^200^

### B. Rethinking Regulation: The Public Utility Model

Can the Public Utility Model be revived for curbing “unfair and oppressive practices in the medical marketplace”?^201^ As Annas has observed, market ideology began to dominate health policy thinking in the 1970s. Nicholas Bagley writes:

Growing faith in the market in the late 1970s and 1980s, a distrust of the cartelization that could arise from single-industry regulation, the political power of organized medicine, and the rise of managed care all contributed to renewed skepticism about the wisdom of treating the medical industry as a public utility. For medicine, approaches that attempt to leverage market forces—not restrain them—have been dominant for three decades.^202^

Bagley reminds us that economic tools and analyses pushed aside the value of the public utility model as discussion turned to health insurance issues. However, regulators still adopted “a persistent, ongoing practice of using state power to curb unfair and oppressive practices in the medical marketplace.”^203^

Financialization of health care and the ferocious drive for profits by financial firms like PE firms have created systematic adverse events in health care: patients ignored, lives shortened or ended prematurely; suffering imposed; costs of care inflated and acting like a tax on every citizen; and a trend toward concentration and even monopolization.

A revitalized public utility model allows for state control over the excesses of for-profit health care, in particular the financialization of everything by private equity firms. Bagley contends that public utility regulation “retains the basic architecture of the private financing system while asserting state control over the medical industry’s perceived excesses. That such regulation would obviate the need for socialized insurance is no coincidence: public utility regulation has long been understood to preserve a role for the private while attending to the needs of the public.”^204^

Bagley hopes that such a model would make regulators target the “large medical systems that are coming to dominate the health-care landscape.”^205^ The model would slow the trend toward concentration as even nonprofit hospitals shed community mission obligations. His concept foreshadows Elizabeth Warren’s *Corporate Crimes Against Health Care Act*, which offers criminal penalties, disgorgement of compensation due to bad outcomes due to looting, and more.^206^ Is this Bagley’s public utility model in the flesh?

### C. Criminalizing Private Equity: Elizabeth Warren Strikes

Elizabeth Warren’s *Corporate Crimes Against Health Care Act* means business. Lawyers are paying attention to the future of health care compliance as public attention focuses on corporate ownership of health care.^207^ The proposal criminalizes behaviors causing patient death among other features.^208^ It offers six regulatory approaches to private equity bad behavior.^209^

Its three most powerful regulatory tools are as follows. First, it imposes both criminal and civil penalties. The criminal penalty imposes up to six years in prison “for executives who loot health care entities like nursing homes and hospitals, if that looting results in a patient’s death.”^210^ It also authorizes a civil penalty in the form of up to five times a federal claw back of all “compensation, including salaries, issued to private equity and portfolio company executives within a 10-year period before or after an acquired health care firm experiences serious, avoidable financial difficulties due to that looting.”^211^

Second, it blocks federal health program payments to entities that sell or use assets for loan collateral to REITs, reduces the 20% pass-through deduction, and otherwise reduces REIT influence.^212^

Third, it requires transparency. Health care providers receiving federal funding must “publicly report mergers, acquisitions, changes in ownership and control, and financial data, including debt and debt-to-earnings ratios.”^213^ Here we see Miller’s accountability principle in full operation, aiming at transparency in the operations of privatized health care entities.^214^

Fourth, harms of corporatization must be reported to Congress by the Office of Inspector General (OIG).^215^

It is hard to imagine an approach to the predatory behavior of private equity firms backed by lobbyists and billions of dollars that would be more unpopular to this lucrative industry and its sponsors. One can hope, however, that some of the Warren regulatory elements can be forced through Congress notwithstanding this inevitable opposition.

### D. Banning Private Equity Completely from Health Care Settings Where Vulnerable Patients/Residents are Involved

The forest was on fire and all the animals swam across the river to escape the fire. The scorpion could not swim and begged the fox to help him. “Please take me across the river on your back – or I will die.” “I am not a fool” said the fox “you will sting me and I will drown.” “No,” replied the scorpion. “I promise that if you help me – I will not sting you – after all, if you drown then so will I.” “Okay” the fox agreed, and the scorpion climbed on his back. Halfway across the river, at the deepest part – the scorpion stung the fox. “Why did you do that?” the fox shouted. “I just couldn’t help it,” cried the scorpion as they both drowned. “It’s just my nature!”^216^

The metaphor of Patient-Centeredness comes closest to reframing the relationship between the patient and the owners of health care facilities. Fiduciary law puts the vulnerable subject of fiduciary duty in a central locale, allowing disgorgement, use of equitable power of the courts, and a focus on patient rights. We must face the fact that private equity’s tools are hard-wired — all regulatory efforts will generate legal workarounds. Perhaps enough is enough.^217^

## Conclusion

We are in the future that Annas, Miller, and Mariner foresaw and worried about in much of their scholarly writing. Health care today is more concentrated, privatized, financialized, and expensive than it was when these giants of health law were writing. The U.S. health care system has become a global embarrassment.^218^ The 2024 Commonwealth Fund Report, comparing ten nations, describes the U.S. system as “failing.”^219^ The failure of the government to regulate financialization has contributed to this sad state of our health care system.

Excellent patient care is available to those of us with wealth or top-notch private employment-based insurance — but employment-based insurance benefits costs consume more and more of employee income. Costs are skyrocketing. Health care institutions are becoming ever more concentrated, and nonprofit missions are shriveling. Quality indicators are improving in some ways, with AI and machine learning tools sources potential improvement, but adoption by hospitals is slow. Far too much of end-of-life care, from nursing homes to hospices, has been seized by the aggressive roll-ups of such entities all over the United States, leaving residents of these institutions facing short staffing, overmedicating, and profound loss of dignity in old age.

We must do better. It is my hope that we health law scholars, both in our scholarship and our advocacy, can facilitate the use of private law to build a model of fiduciary duties on health care institutions, and, in Nicolas Bagley’s words, make better use of “state power to curb unfair and oppressive practices in the medical marketplace.”^220^ We can also better educate our law students to understand the economics of the new health care world, and make better choices about what to do and who to represent.

## Footnotes

1 These books and articles include George J. Annas, Some Choice: Law, Medicine, and the Market (1998); Francis H. Miller, *Health Care Information Technology and Provider Accountability: A Symbiotic Relationship*, *in* Law and Medicine: Current Legal Issues (Michael Freeman & Andrew Lewis eds., 2000); and Wendy K. Mariner, *Did Legal Education Fail Health Reform? And How Health Law Can Help*, 10 Ind. L. Rev. 1 (2013).

2 Annas, *supra* note 1, at 48.

3 Miller, *supra* note 1, at 28.

4
*Id.* at 29.

5 Henk ten Have & Bert Gordijn*, Metaphors in Medicine*, 25 Med. Health Care & Phil. 577, 577 (2022) (internal citations omitted).

6
*See generally* Ctrs. for Medicare & Medicaid Servs., History of Health Spending in the United States, 1960-2013 (2015).

7
*See generally* Ester Uyehara & Margaret Thomas, Health Maintenance Organization and the HMO Act of 1973 (1975).

8
*See generally* Annas, *supra* note 1.

9
*See* George J. Annas, *Reframing the Debate on Health Care Reform by Replacing Our Metaphors*, 332 New Eng. J. Med. 744, 745 (1995).

10
*See id.*

11 Paul Hodgkin, *Medicine Is War: And Other Medical Metaphors*, 291 Brit. Med. J. 1820, 1820 (1985).

12
*See id.* at 1821.

13
*See id.*

14 Annas, *supra* note 1, at 46.

15 Bernard M. Dickens, *George J. Annas, Some Choice: Law, Medicine, and the Market. New York and Oxford University Press, 1998*, 15 J. Contemp. Health L. & Pol’y 609, 613 (1999) (book review).

16
*Id.*; *see also generally* John Coggon, *What Help Is a Steward? Stewardship, Political Theory, and Public Health Law and Ethics*, 62 N. Ire. Q. 599 (2011) (discussing the critical treatment of “stewardship” generally as a concept in the health care area).

17 Barry R. Furrow, *The Role of the Lawyer as Deal Maker in Health Care Acquisitions: From Amoral to Immoral?*, 52 J.L. Med. & Ethics 333, 335 (2024).

18
*Id.*

19
*Id.*

20
*See* Mike Hill & Warren Montag, *The Economic Imagination: The Magical Thinking of Economists Is a Pathology of Thought with Deep Historical Roots*, Stan. Univ. Press Blog (Aug. 20, 2015), https://stanfordpress.typepad.com/blog/2015/08/the-economic-imagination-.html [https://perma.cc/F4AL-SU8Q]; Adam Hayes, *Perfect Competition: Examples and How It Works*, Investopedia (Sept. 26, 2024), https://www.investopedia.com/terms/p/perfectcompetition.asp (describing the theoretical assumptions required for perfect competition).

21
*See generally* Lee Boldeman, The Cult of the Market: Economic Fundamentalism and Its Discontents (2007).

22
*See generally* Leslie King, *How Government Created and Shaped the U.S. Nursing Home Industry*, 46 Critical Socio. 881 (2020).

23 Furrow, *supra* note 17, at 335; Itai Green, *Innovation in Focus: Private Equity and Institutional Actors*, Innovation Mgmt. (Apr. 1, 2021), https://innovationmanagement.se/2021/04/01/innovation-in-focus-private-equity-and-institutional-actors/ [https://perma.cc/ES9W-UBPN].

24 Glenn Barenbaum & Lance Beder, *The Growing Role of Private Equity in Healthcare*, GrantThornton (Sept. 26, 2023), https://www.grantthornton.com/insights/articles/health-care/2023/the-growing-role-of-private-equity-in-healthcare [https://perma.cc/8E34-T4EL].

25 Furrow, *supra* note 17, at 335.

26 I have argued that private equity’s imagined innovation tools when rolling up behavioral health facilities was in part based on the promise of AI-driven behavioral therapies using avatars and other AI tools in place of living therapists. *See generally* Barry R. Furrow, *The Future of Behavioral Health: Can Private Equity and Telehealth Improve Access?*, 49 Am. J.L. & Med. 314 (2023).

27 Furrow, *supra* note 17, at 335.

28
*Id.*

29
*Id.*; *see also Private Equity Ownership of Nursing Homes Linked to Lower Quality of Care, Higher Medicare Costs*, Weill Cornell Med. (Nov. 19, 2021), https://news.weill.cornell.edu/news/2021/11/private-equity-ownership-of-nursing-homes-linked-to-lower-quality-of-care-higher.

30 Furrow, *supra* note 17, at 335; *see also* Hayes, *supra* note 20.

31
*See* Furrow, *supra* note 17, at 335.

32
*Id.*

33
*Id.*

34
*Id.*; The power of private equity in so-called middle markets — retail products, software and technology products, and other elements of an industrial economy — may well achieve benefits for consumers. The risk of financial distress is much less in these middle markets. It is leveraged buyouts and other strategies in the private equity playbook that create financial distress, bankruptcies, and suffering for nursing home residents. *See generally* Robert I. Field et al., *Private Equity in Health Care: Barbarians at the Gate?*, 15 Drexel L. Rev. 821 (2023).

35 Fernand Braudel, The Wheels of Commerce 230 (Sian Reynolds ed., 1983).

36
*See* Donald Cohen, *The Roots and Reasons of Privatization*, The Forge (May 5, 2022), https://forgeorganizing.org/article/roots-and-reasons-privatization [https://perma.cc/E4JN-FJH6]. Cohen argues that the advocates of privatization are imbedded in our national politics—“images of lazy, unionized bureaucrats; looming bureaucracies that are remarkably both inefficient and nefarious; and the utopian promise of competition.” *Id.*; *see also* Exec. Order No. 12,607, 52 Fed. Reg. 34190 (Sept. 9, 1987) (establishing Reagan’s Commission on Privatization).

37 Laura B. Gardner & Richard M. Scheffler, *Privatization in Health Care: Shifting the Risk*, 45 Med. Care Rsch. & Rev. 215, 216 (1988).

38 Benjamin Goodair & Aaron Reeves*, The Effect of Health Care Privatisation on the Quality of Care*, 9 Lancet Pub. Health e199, e199 (2024).

39
*Id.*

40 Cohen, *supra* note 36.

41 Furrow, *supra* note 17, at 335; *See* John B. Goodman & Gary W. Loveman, *Does Privatization Serve the Public Interest?*, Harv. Bus. Rev. (1991), https://hbr.org/1991/11/does-privatization-serve-the-public-interest.

42 Goodair & Reeves, *supra* note 38.

43
*Id.*

44
*Id.* at e205.

45
*See* King, *supra* note 22, at 891.

46
*See* Eileen Appelbaum & Rosemary Batt, *Financialization in Health Care: The Transformation of US Hospital Systems* 53–77 (Ctr. for Econ. & Pol’y Rsch., Working Paper, Sept. 9, 2021), https://www.cepr.net/wp-content/uploads/2021/10/AB-Financialization-In-Healthcare-Spitzer-Rept-09-09-21.pdf [https://perma.cc/98N9-VL63].

47 Hannah Levintova, *The Smash-and-Grab Economy*, Mother Jones, May-June 2022, at 14, 17, https://www.motherjones.com/politics/2022/05/private-equity-buyout-kkr-houdaille/ [https://perma.cc/9PU2-S6LD].

48
*See generally* Gerald F. Davis & Suntae Kim, *Financialization of the Economy*, 41 Ann. Rev. Socio. 203 (2015).

49
*See Financialization: Definition, Examples, Consequences, and Criticisms*, Investopedia (Aug. 23, 2024), https://www.investopedia.com/terms/f/financialization.asp [https://perma.cc/A5C5-669F] (defining financialization as “increase in size and importance of a country’s sector relative to its overall economy”).

50 Brendan Ballou, *When Private-Equity Firms Bankrupt Their Own Companies*, Atlantic (May 1, 2023), https://www.theatlantic.com/ideas/archive/2023/05/private-equity-firms-bankruptcies-plunder-book/673896/ [https://perma.cc/4QK4-KQH3].

51 My research on private equity in health care includes the following materials: Furrow, *supra* note 17, at 336; Furrow, *supra* note 26, at 328; Robert I. Field et al., *supra* note 34; Barry R. Furrow, *The Hollowed-Out American Nursing Home: Using Private Law to Police Poor Quality Care and Expand Owner Responsibilities*, *in* Health Law as Private Law (I. Glenn Cohen et al. eds., 2025) [hereinafter *Hollowed-Out*].

52 Fred Schulte, *Sick Profit: Investigating Private Equity’s Stealthy Takeover of Health Care Across Cities and Specialties*, Kaiser Fam. Found. Health News (Nov. 14, 2022), https://kffhealthnews.org/news/article/private-equity-takeover-health-care-cities-specialties/ [https://perma.cc/8F79-ZY85].

53
*Id.*

54 Appelbaum & Batt, *supra* note 46, at 1.

55
*Private Equity Industry Overview*, St. of Walls (2013), https://www.streetofwalls.com/finance-training-courses/private-equity-training/private-equity-industry-overview/ [https://perma.cc/WPU6-2UER].

56
*See* Appelbaum & Batt, *supra* note 46, at 58; *see also id.* at 70–74 (discussing instances of private equity firms selling healthcare properties at the patients’ expense).

57 Will Kenton, *Leveraged Buyout (LBO): Definition, How It Works, and Examples*, Investopedia (June 8, 2024), https://www.investopedia.com/terms/l/leveragedbuyout.asp [https://perma.cc/N9RJ-KNM9].

58
*Id.*

59
*Id.*

60 Furrow, *supra* note 26, at 334; *see also* Eileen O’Grady, *Private Equity Firms Reaped Billions of Dollars in Debt-Funded Dividends from Healthcare Companies in 2021*, Priv. Equity Stakeholder Project (Jan. 5, 2022), https://pestakeholder.org/news/private-equity-firms-reaped-billions-of-dollars-in-debt-funded-dividends-from-healthcare-companies-in-2021/ [https://perma.cc/4W7F-ACLA].

61
*See Hollowed-Out*, *supra* note 51, at 273; Elizabeth Schrier et al., *Hospital Assets Before and After Private Equity Acquisition*, 332 J. Am. Med. Ass’n 669 (2024).

62
*See* O’Grady, *supra* note 60.

63 Jordan Rau, *Nursing Home Owners Drained Cash While Residents Deteriorated, State Filings Suggest*, NPR (Jan. 31, 2023), https://www.npr.org/sections/health-shots/2023/01/31/1139783599/new-york-nursing-home-owners-drained-cash [https://perma.cc/ZES2-YKK4].

64
*See* Brian Ayash & Mahdi Rastad, *Leveraged Buyouts and Financial Distress*, Fin. Rsch. Letters, Jan. 22, 2021, at 1, 4–5.

65
*See Hollowed-Out*, *supra* note 51, at 273.

66
*Id.*; *see also* Field et al., *supra* note 34, at 858–62; David Brancaccio & Jarrett Dang, *Private Equity Bought a Nursing Home, Leading to Staff Cuts and a Decline in Care*, Marketplace (Sept. 8, 2022), https://www.marketplace.org/story/2022/09/08/private-equity-bought-a-nursing-home-leading-to-staff-cuts-and-a-decline-in-care.

67 Goodair & Reeves, *supra* note 38, at e201.

68 Mark Duggan et al., *The Impact of Privatization: Evidence from the Hospital Sector* 3, 21, 30, 35 (Nat’l Bureau of Econ. Rsch., Working Paper No. 30824, 2023), https://www.nber.org/system/files/working_papers/w30824/w30824.pdf [https://perma.cc/3FNH-5GLC].

69
*Hollowed-Out*, *supra* note 51, at 274.

70
*Id.* at 274; *see also* Melea Atkins, *The Impact of Private Equity on Nursing Home Care: Recommendations for Policymakers*, Roosevelt Inst. 6–7 (Apr. 2021), https://rooseveltinstitute.org/wp-content/uploads/2021/04/RI_NursingHomesandPE_IssueBrief_202104.pdf [https://perma.cc/Y254-AUYV].

71 Jordan Rau, *Care Suffers as More Nursing Homes Feed Money into Corporate Webs*, N.Y. Times (Jan. 2, 2018), https://www.nytimes.com/2018/01/02/business/nursing-homes-care-corporate.html [https://perma.cc/5K8R-ZDB6].

72
*Id.*

73
*See* Appelbaum & Batt, *supra* note 46, at 38–40; *Commercial Real Estate Investor & Private Equity Liability*, First Nat’l Realty Partners (Mar. 2, 2022), https://fnrpusa.com/blog/commercial-real-estate-investor-private-equity-liability/ [https://perma.cc/KE2D-JKWZ].

74
*Hollowed-Out*, *supra* note 51, at 274.

75
*Id.*; *see also* Yao O. Dinizulu & Jennifer Matta, *The Multi-Level Nursing Home Corporate Structure: Transparency, Accountability and Common Sense*, 12 Andrews Nursing Home Litig. Rep. 1, 2–3 (2009).

76
*Id.*; David E. Kingsley & Charlene Harrington, *Financial and Quality Metrics of a Large, Publicly Traded U.S. Nursing Home Chain in the Age of Covid-19*, 52 Int’l J. Health Servs. 212, 221 (2022).

77 Miller, *supra* note 1, at 28.

78
*Id.*

79
*Id.*

80
*Id.*

81
*Id.*

82
*Id.* at 29.

83
*See generally* Aaron D. Twerski & Neil B. Cohen, *Comparing Medical Providers: A First Look at the New Era of Medical Statistics*, 58 Brook. L. Rev. 5 (1992); Jesse Green, *Problems in the Use of Outcome Statistics to Compare Health Care Providers*, 58 Brook. L. Rev. 55 (1992).

84
*See generally* Frances H. Miller, *Medical Discipline in the Twenty-First Century: Are Purchasers the Answer?*, 60 L. & Contemp. Probs. 31 (1997).

85 Barry R. Furrow, *The Limits of Current A.I. in Health Care: Patient Safety Policing in Hospitals*, 12 Ne. U. L. Rev. 1, 27 (2020).

86
*Id.*; *see also Focused Professional Practice Evaluation (FPPE) - Understanding the Requirements*, The Joint Comm’n (May 10, 2022), https://www.jointcommission.org/standards/standard-faqs/critical-access-hospital/medical-staff-ms/000001485/ [https://perma.cc/C2AH-K39T].

87 “The Joint Commission (TJC) is an independent, not-for-profit organization created in 1951 that accredits more than 20,000 US health care programs and organizations.” Roopma Wadhwa & Annie P. Boehning, *The Joint Commission*, StatPearls Publ’g 1, 1 (2024), https://www.ncbi.nlm.nih.gov/books/NBK557846/ [https://perma.cc/RAG7-9XQ7]. Its primary focus is on patients and patient safety harms. The Joint Commission states that its mission is “[t]o continuously improve health care for the public, in collaboration with other stakeholders, by evaluating health care organizations and inspiring them to excel in providing safe and effective care of the highest quality and value.” *Id.*

88 Furrow, *supra* note 85, at 27.

89
*Id.*

90
*Id.*

91 Barry R. Furrow, *The Confused and Bewildered Hospital: Adverse Event Discovery, Pay-for-Performance, and Big Data Tools as Halfway Technologies*, 46 Am. J.L. & Med. 219, 233 (2020).

92 Ziad Obermeyer & Ezekiel J. Emanuel, *Predicting the Future – Big Data, Machine Learning, and Clinical Medicine*, 375 New Eng. J. Med. 1216, 1217 (2016).

93 42 C.F.R. § 482.12(a)(5) (2024).

94 See Barry R. Furrow*, Eleanor Kinney Tribute*, 17 Ind. Health L. Rev. 79, 80 (2020).

95
*Id.* at 81.

96
*See* Furrow, *supra* note 85, at 29–31.

97 See Eleanor D. Kinney, *The Accidental Administrative Law of the Medicare Program*, 15 Yale J. Health Pol’y L. & Ethics 111, 115 (2015).

98
*Artificial Intelligence*, Healthcare Dive, https://www.healthcaredive.com/trendline/artificial-intelligence/102/ [https://perma.cc/C9KX-632V] (last visited Mar. 7, 2025).

99 Dylan Thomas, *Private Equity Bets on AI Gold Rush with Billions Pumped into Datacenters*, S & P Glob. (Dec. 11, 2023), https://www.spglobal.com/marketintelligence/en/news-insights/latest-news-headlines/private-equity-bets-on-ai-gold-rush-with-billions-pumped-into-datacenters-79666528 [https://perma.cc/B5FS-SAJN].

100
*See generally* Mariner, *supra* note 1, at 1.

101
*See id.* at 2.

102
*Id.* at 21.

103
*Id.* at 25.

104
*Id.* (quoting George J. Annas, Standard of Care: The Law of American Bioethics 246–49 (1993)).

105 Rau, *supra* note 71.

106
*See* Ayash & Rastad, *supra* note 64, at 5.

107 This section is drawn from my article Furrow, *supra* note 17.

108 Schenck v. Living Centers-East, Inc., 917 F. Supp. 432, 437–38 (E.D. La. 1996).

109
*Hollowed-Out*, *supra* note 51, at 278; *see also* J. Boldt, *The Concept of Vulnerability in Medical Ethics and Philosophy*, 14 Phil. Ethics & Humans. Med. 1 (2019); D. Gordon Smith, *The Critical Resource Theory of Fiduciary Duty*, 55 Vand. L. Rev. 1399, 1404 (2022).

110
*Hollowed-Out, supra* note 51, at 278 (quoting Petre v. Living Centers-East, 935 F. Supp. 808, 812 (E.D. La. 1996)).

111
*Id.* at 279.

112
*Id.*; *see also* Leonard I. Rotman, *Understanding Fiduciary Duties and Relationship Fiduciarity*, 62 McGill L.J. 975, 1005 (2017).

113
*Hollowed-Out*, *supra* note 51, at 279.

114 Julian Velasco, *Fiduciary Judgment Rules*, 62 Wm. & Mary L. Rev. 1397, 1405 (2021), https://scholarship.law.wm.edu/wmlr/vol62/iss4/8 [https://perma.cc/RH6S-83T2] (quoting Samuel L. Bray, *Fiduciary Remedies*, *in* The Oxford Handbook of Fiduciary Law 449, 449–50 (Evan J. Criddle, Paul B. Miller & Robert H. Sitkoff eds., 2019)).

115 Furrow, *supra* note 17, at 339.

116
*Id.* at 343.

117
*Id.*

118
*Id.*

119
*Id.*

120
*Id.*

121
*Hollowed-Out*, *supra* note 51, at 279; *see also* Kingsley & Harrington, *supra* note 76.

122
*Hollowed-Out*, *supra* note 51, at 279.

123
*Id.*; *see also* O’Grady, *supra* note 60.

124 For an excellent history of developments in the model rules as the legal profession changed and fractured, see generally Michael S. Ariens, *The Agony of Modern Legal Ethics, 1970–1985*, 5 St. Mary’s J. on Legal Malpractice & Ethics 134 (2014).

125 Furrow, *supra* note 17, at 342; Model Rules of Pro. Conduct r. 1.6 (Am. Bar Ass’n 1983).

126 Model Rules of Pro. Conduct preface (Am. Bar Ass’n 1983).

127 Furrow*, supra* note 17, at 342.

128 Model Rules of Pro. Conduct r. 1.6 (Am. Bar Ass’n 1983).

129 Furrow, *supra* note 17, at 342.

130
*Id.*

131
*Id.*

132 Model Rules of Pro. Conduct r. 1.2(d) (Am. Bar Ass’n 1983).

133
*Id.* r. 4.1(b).

134
*See also* Katerina P. Lewinbuk, *Keep Suing All the Lawyers: Recent Developments in Claims Against Lawyers for Aiding & Abetting a Client’s Breach of Fiduciary Duty*, 8 St. Mary’s J. on Legal Malpractice & Ethics 158, 158–79 (2018).

135 Furrow, *supra* note 17, at 342.

136
*Id.*; *see also* Lewinbuk, *supra* note 134, at 163 (noting twenty-three states have adopted this theory); Deborah A. DeMott, *Beyond Metaphor: An Analysis of Fiduciary Obligation*, 5 Duke L.J. 879, 879 (1988) (characterizing fiduciary liability as “situation-specific” depending on parties and relationships).

137 Restatement (Second) of Torts § 874 (Am. L. Inst. 1979).

138 Restatement (Third) of the Law Governing Lawyers § 51 (Am. L. Inst. 2000).

139
*See* Furrow, *supra* note 17, at 342.

140 Restatement (Third) of the Law Governing Lawyers § 51 cmt. H (Am. L. Inst. 2000); *see also* Leroy v. Allen, 872 N.E. 2d 254, 258 (Ohio 2007) (“[A]n attorney retained by a fiduciary owes a similar duty to those with whom the client has a fiduciary relationship.”); Brinkley Rowe, *See No Fiduciary, Hear No Fiduciary: A Lawyer’s Knowledge Within Aiding and Abetting Fiduciary Breach Claims*, 85 Fordham L. Rev. 1389, 1397 (2016).

141
*See* Furrow, *supra* note 17, at 342–43.

142 Restatement (Second) of Torts § 876 (Am. L. Inst. 1979).

143 Furrow, *supra* note 17, at 344; *see also generally* Erin C. Fuse Brown & Mark A. Hall, *Private Equity and the Corporatization of Health Care*, 76 Stan. L. Rev. 527 (2024).

144 Furrow, *supra* note 17, at 344–45; *see also* Doreen McBarnet, *Financial Engineering or Legal Engineering? Legal Work, Legal Integrity and the Banking Crisis* (Univ. of Edinburgh Sch. of L., Working Paper No. 02, 2010).

145
*See* Furrow, *supra* note 17, at 345.

146
*Id.*

147 Perhaps the model from another angle is the Antonin Scalia Law School at George Mason University, committing to advancing law and economics as a tool of the right.

148
*Remembering Edgar Cahn: Social Justice Champion and Co-Founder of UDC Law’s Predecessor School*, Univ. of D.C. Sch. of L. (Feb. 3, 2022), https://law.udc.edu/2022/02/03/remembering-edgar-cahn-social-justice-champion-and-co-founder-of-udc-laws-predecessor-school/ [https://perma.cc/DDX7-D2J7].

149
*Id.*

150 Steven Waldman, *’A Perfect Combination of Chutzpah and Soul*,*’* Wash. Post. (Aug. 17, 1991), https://www.washingtonpost.com/archive/lifestyle/magazine/1991/08/18/a-perfect-combination-of-chutzpah-and-soul/2b1f9269-3b44-45ab-9b5b-19e70c5f7773/.

151 Emily Langer, *Edgar Cahn, Humanitarian Who Championed Legal Services for Poor, 86*, Bos. Globe, Jan. 30, 2022, at A25.

152 Univ. of D.C. Sch. of L., *supra* note 148.

153 Nora Freeman & James Stone, *Auto Clubs and the Lost Origins of the Access-to-Justice Crisis*, 134 Yale L.J. 123, 127 (2024).

154
*Id.* at 123.

155
*Id.* at 171.

156
*Id.* at 127–28.

157 Donald M. Berwick, *What ‘Patient-Centered’ Should Mean: Confessions of an Extremist*, 28 Health Affs. w555, w564 (2009) (emphasis added).

158 Annas, *supra* note 9, at 746.

159
*Id.*

160
*Id.*

161
*Id.* at 147.

162 Anne Clancy et al., *The Meaning of Dignity for Older Adults: A Meta-Synthesis*, 28 Nursing Ethics 878, 879 (2021).

163
*Id.*

164
*Id.*

165
*Id.*

166
*Id.*

167 Robert Brenson et al., *Health Care Stewardship Case Studies*, Urb. Inst, https://www.urban.org/policy-centers/health-policy-center/projects/health-care-stewardship-case-studies [https://perma.cc/4QNS-3BVQ] (last visited Mar. 6, 2025).

168
*Id.*

169
*Id.*

170
*See* Coggon, *supra* note 16, at 613 (criticizing the concept of “stewardship”).

171
*See* Brenson et al., *supra* note 167.

172
*Id.*

173 David Gorski, *Dr. Donald Berwick and “Patient-Centered” Medicine: Letting the Woo into the New Health Care Law?*, Sci.-Based Med. (July 12, 2010), https://sciencebasedmedicine.org/dr-donald-berwick-and-patient-centered-medicine-letting-the-woo-into-the-new-health-care-law/ [https://perma.cc/2SKD-WJE6].

174
*See generally* George J. Annas & Joseph M. Healey, Jr., *The Patient Rights Advocate: Redefining the Doctor-Patient Relationship in the Hospital Context*, 27 Vand. L. Rev. 243, 257–65 (1974).

175
*Id.* at 257–68.

176 Furrow, *supra* note 26, at 337; *see also generally Hollowed-Out*, *supra* note 51.

177 Corporate Crimes Against Health Care Act, S. 4503, 118th Cong. (2024).

178
*See, e.g.*, Elisabeth Harrison & Roberto Scalese, *A Timeline of Steward Health Care, from Founding to Financial Peril*, WBUR (Jan. 31, 2024), https://www.wbur.org/news/2024/01/31/steward-health-care-timeline [https://perma.cc/Z9YX-T5EN]; Reuters, *Bankrupt Steward Health Puts Its Hospitals up for Sale, Discloses $9 Billion in Debt*, CNN Bus. (May 7, 2024), https://www.cnn.com/2024/05/07/business/steward-health-puts-hospitals-up-for-sale/index.html [https://perma.cc/XB5Q-SK6A]; Corporate Crimes Against Health Care Act, S. 4503, 118th Cong. (2024).

179 I use these metaphors to capture one version of regulation that polices behavior by slowing it or penalizes excessive behavior that risks harms to others. Speed bumps are raised areas on roads that force a driver to slow to avoid harming a car; spike strips will stop speeding by puncturing driver tires so they must stop; and radar guns are a tool to enable police to enforce traffic speed laws. These tool analogies have some useful value in framing and visualizing regulation.

180 Furrow, *supra* note 26, at 336. I, along with other scholars, considered the merits of banning private equity firms from owning nursing homes, given the vulnerability of residents. *See generally* Field et al., *supra* note 34, at 885.

181 Furrow, *supra* note 26, at 336–37; *see also* Emily Gee & Thomas Waldrop, *Policies to Hold Nonprofit Hospitals Accountable*, Ctr. for Am. Progress (Oct. 18, 2022), https://www.americanprogress.org/article/policies-to-hold-nonprofit-hospitals-accountable/.

182 Furrow, *supra* note 26, at 337.

183
*Id.* at 329; *see also* Atul Gupta et al., *Owner Incentives and Performance in Healthcare: Private Equity Investment in Nursing Homes* 2–3, 72 tbl.C.3 (Nat’l Bureau of Econ. Rsch., Working Paper No. 28474, 2021), http://www.nber.org/papers/w28474 [https://perma.cc/7E2M-ZKLG].

184 Gupta, *supra* note 183, at 2–3, 72 tbl.C.3.

185
*See* U.S. Gov’t Accountability Off., GAO-22-105133, COVID-19 in Nursing Homes: CMS Needs to Continue to Strengthen Oversight of Infection Prevention and Control (2022); U.S. Gov’t Accountability Off., GAO-19-433, Nursing Homes: Improved Oversight Needed to Better Protect Residents from Abuse (2019).

186 Nori Gerardo Lietz & Zirui Song, *Does Private Equity Have Any Business Being in the Health Care Business?* (Harv. Bus. Sch., Working Paper No. 25-012, 2024).

187
*Id.* at 9.

188 Furrow, *supra* note 26, at 337; *see also* Mary Bugbee et al., Recent Trends in Private Equity Healthcare Acquisitions 15–16 (2023).

189 Furrow, *supra* note 26, at 337; *see also* Bugbee et al., *supra* note 188, at 15–16.

190 Furrow, *supra* note 26, at 337; *see also* Bugbee et al., *supra* note 188, at 15–16.

191 Furrow, *supra* note 26, at 337; *see also* Bugbee et al., *supra* note 188, at 15–16.

192 Furrow, *supra* note 26, at 337; *see also* Bugbee et al., *supra* note 188, at 16–17.

193 Bugbee et al., *supra* note 188, at 17; *see also* Furrow, *supra* note 26, at 337.

194 Alexandra D. Montague et al., State Action to Oversee Consolidation of Health Care Providers 11 (August 2021), https://www.milbank.org/wp-content/uploads/2021/08/State-Action-to-Oversee-Consolidation_ib_V3.pdf [https://perma.cc/VXQ2-XQEE]; *see also* Furrow, *supra* note 26, at 337.

195 Furrow, *supra* note 26, at 337.

196 Furrow, *supra* note 26, at 337. As noted in this previous article, since 2013, at least 25 private equity-backed health care companies have paid settlements in excess of $570 million for allegedly violations of the FCA. Eileen O’Grady, Priv. Equity Stakeholder Project, Money for Nothing 2 (2021), https://pestakeholder.org/wp-content/uploads/2021/02/Private-Equity-False-Claims-Act-PESP-022221-.pdf. Behavioral health companies may be at greater risk for enforcement (compared to other health care services companies) given the availability of legislative tools specific to behavioral health, such as EKRA, the Eliminating Kickbacks in Recovery Act of 2018*. See, e.g.*, Sean J. Quinn, *Private Equity in Behavioral Health: Clinical Due Diligence Requires Consideration of EKRA, the New Anti-Kickback Law Applicable to All Payors*, Husch Blackwell (Mar. 28, 2019), https://www.healthcarelawinsights.com/2019/03/private-equity-in-behavioral-health-clinical-due-diligence-requires-consideration-of-ekra-the-new-anti-kickback-law-applicable-to-all-payors/.

197 Furrow, *supra* note 26, at 338; *see also Hollowed-Out*, *supra* note 51, at 271 (using corporate negligence and fiduciary obligation as a foundation to “build a private law doctrinal framework to better police the waste, fraud, and pillaging of assets by PE owners of nursing home systems”).

198 Furrow, *supra* note 26, at 338.

199 16 C.F.R § 314 (2023) (creating “standards for developing, implementing, and maintaining reasonable administrative, technical, and physical safeguards to protect the security, confidentiality, and integrity of customer information”); *see also FTC Safeguards Rule: What Your Business Needs to Know*, Fed. Trade Comm’n (Dec. 2024), https://www.ftc.gov/business-guidance/resources/ftc-safeguards-rule-what-your-business-needs-know [https://perma.cc/S8Y9-DARL].

200
*See generally* David Dayen, *Long-Awaited Rules on Private Equity Mostly Involve Disclosure*, Am. Prospect (August 25, 2023), https://prospect.org/economy/2023-08-25-private-equity-rules-mostly-involve-disclosure.

201 Nicholas Bagley, *Medicine as a Public Calling*, 114 Mich. L. Rev. 57, 61 (2015).

202
*Id.* at 99–100.

203
*Id.* at 61.

204
*Id.* at 70.

205
*Id.* at 101.

206 Corporate Crimes Against Health Care Act, S. 4503, 118th Cong. (2024).

207
*See, e.g.*, John C. Saran et al., *Healthcare Private Equity Transactions Under Scrutiny: Midyear Review*, Holland & Knight (Aug. 1, 2024), https://www.hklaw.com/en/insights/publications/2024/08/healthcare-private-equity-transactions-under-scrutiny-midyear-review [https://perma.cc/DUX2-7GVM].

208 Corporate Crimes Against Health Care Act, S. 4503, 118th Cong. (2024); Press Release, United States Senator for Massachusetts, Senators Markey, Warren Introduce the Corporate Crimes Against Health Care Act of 2024 (June 11, 2024) [hereinafter Markey Press Release], https://www.markey.senate.gov/news/press-releases/senators-markey-warren-introduce-the-corporate-crimes-against-health-care-act-of-2024 [https://perma.cc/9NMZ-VJSV].

209 Corporate Crimes Against Health Care Act, S. 4503, 118th Cong. (2024). Warren summarizes the six regulatory provisions of the Corporate Crimes Against Health Care Act as follows: It will (1) “Create a new criminal penalty of up to 6 years in prison for executives who loot health care entities like nursing homes and hospitals, if that looting results in a patient’s death”; (2) “Provide state attorneys general and the DOJ with the power to claw back all compensation, including salaries, issued to private equity and portfolio company executives within a 10- year period before or after an acquired health care firm experiences serious, avoidable financial difficulties due to that looting”; (3) “Authorize an associated civil penalty of up to 5 times the clawback amount;” (4) “Prohibit payments from federal health programs to entities that sell assets or use assets for a loan collateral made to a REIT, with an exemption for current arrangements; repeal a rule in the Tax Code that allows taxable REIT subsidiaries to exert influence on the operations of health care entities; and remove the 20 percent pass-through deduction, passed in the 2017 Trump tax cuts, for all REIT investors”; (5) “Require health care providers receiving federal funding to publicly report mergers, acquisitions, changes in ownership and control, and financial data, including debt and debtto-earnings ratios”; (6) “Mandate an HHS OIG report to Congress on the harms of corporatization in health care.” Elizabeth Warren, *The Corporate Crimes Against Health Care Act of 2024*, U.S. Sen. Elizabeth Warren, https://www.warren.senate.gov/imo/media/doc/Corporate%20Crimes%20Against%20Health%20Care%20Act%20one-pager.pdf [https://perma.cc/DZL8-NP8S] (last visited June 19, 2025).

210 Markey Press Release, *supra* note 208.

211
*Id.*

212
*Id.*

213
*Id.*

214 Miller, *supra* note 1, at 73.

215 Markey Press Release, *supra* note 208.

216 Furrow, *supra* note 26, at 327 (citing @valeriamedina1611, *The Fox and The Scorpion*, Scribd, https://www.scribd.com/document/767076839/The-fox-and-the-scorpion (last visited June 17, 2025)).

217
*See* discussion *supra* Section V.A.

218
*See generally* David Blumenthal et al., Commonwealth Fund, Mirror, Mirror 2024: A Portrait of the Failing U.S. Health System (2024) (comparing the U.S. health system with health systems in nine other countries across seventy health system performance measures).

219
*Id.* at 14 (“Despite spending a lot on health care, the United States is not meeting one of the principal obligations of a nation: to protect the health and welfare of its residents. Most of the countries we compared are providing this protection, even though each can learn a good deal from its peers. The U.S., in failing this ultimate test of a successful nation, remains an outlier.”).

220
*See* Bagley, *supra* note 201, at 61.

